# A new commercial boundary dataset for metropolitan areas in the USA and Canada, built from open data

**DOI:** 10.1038/s41597-024-03275-3

**Published:** 2024-04-24

**Authors:** Byeonghwa Jeong, Jeff Allen, Karen Chapple

**Affiliations:** 1 Postdoctoral Fellow, School of Cities, University of Toronto, Toronto, Canada; 2 Lead, Data Visualization, School of Cities, University of Toronto, Toronto, Canada; 3 Director, School of Cities and Professor, Geography & Planning, University of Toronto, Toronto, Canada

**Keywords:** Geography, Society, Business

## Abstract

The purpose of this study is to define the geographic boundaries of commercial areas by creating a consistent definition, combining various commercial area types, including downtowns, retail centres, financial districts, and other employment subcentres. Our research involved the collection of office, retail and job density data from 69 metropolitan regions across USA and Canada. Using this data, we conducted an unsupervised image segmentation model and clustering methods to identify distinctive commercial geographic boundaries. As a result, we identified 23,751 commercial areas, providing a detailed perspective on the commercial landscape of metropolitan areas in the USA and Canada. In addition, the generated boundaries were successfully validated through comparison with previously established commerce-related boundaries. The output of this study has implications for urban and regional planning and economic development, delivering valuable insights into the overall commercial geography in the region. The commercial boundary and used codes are freely available on the School of Cities Github, and users can reuse, reproduce and modify the boundaries.

## Background & Summary

Commercial areas - what, where, and how do we define them? Despite the consistent demand to create a generalisable definition of commercial areas from researchers, government agencies, and related stakeholders, the related studies tend to focus on specific types, such as retail agglomerations^1,2^, or functions like downtown regions^3,4^, Commercial Centres (CCs) and Central Business Districts (CBDs)^5^. However, commercial activities are not limited to just retail and specific functions, meaning a more consistent perspective could facilitate a holistic understanding of these commercialised zones. Consequently, the aim of this study is to establish a unified definition for commercial areas and to capture their boundaries accordingly.

To develop this definition, we explored areas associated with commerce such as downtowns, main streets, and retail centres. A downtown, generally understood as the central area or CBD of cities, typically has a concentration of commercial, entertainment, and cultural activities^6^. However, the notion of ‘downtown’ is subjective and differs based on individual perceptions^3^, making it challenging to establish exact boundaries, particularly in comparative studies. To tackle this ambiguity, an alternative approach is to employ the degree of concentration of jobs. For example, downtown areas developed by the Brookings Institution^5^ as well as the University of Toronto’s Downtown Recovery project^4^ defines and identifies the downtown based on the job density.

In terms of retail agglomeration, it can be seen that there are two types of retail areas, namely the unplanned commercial districts (e.g. commercial districts, main streets, high streets, etc.) and the planned shopping centres (e.g. shopping malls, retail parks, etc.)^7^. Unplanned commercial districts can be defined as areas that evolved naturally by agglomerating retailers. Primarily local residents patronise these areas, and the retailers locate in an ad hoc, unplanned manner^7–10^. Planned shopping centres can be considered another type of retail agglomerated area. The notable feature of these areas is that they are intentionally developed and managed by large private companies and financial institutions in sub-urban or peri-urban areas based on balanced and planned tenancy. In addition, the planned shopping centres tend to be based on one or a few large-size unified buildings and equipped with large car parks in terms of morphology^7,8,11^.

Lastly, financial and office -districts tend to be considered the main place with commercial activities. The most salient characteristic of this type of area is clusters of the offices and buildings serving diverse professional services associated with business, financial, etc. This area tends to be located in the heart of cities. In the case of metropolitan areas, it is separately located from the retail areas, whereas for smaller cities, financial and office -districts are likely to be located together with the major retail areas^12^.

Synthesizing these characteristics, the definition of commercial areas can be established as a spatial extent where commercial elements - office, retail, and jobs - are aggregated together, thereby covering these diverse definitions of areas related to commercial activities. The definition of job is the number of workers at workplaces regardless of the sort of jobs. The reason for employing all jobs was that it was difficult to define the retail and office-related jobs, and there was a limit to data acquisition. On the basis of this established definition, we conducted a series of analyses in order to identify the spatial range of commercial areas (called commercial boundaries).

To achieve this goal, we selected 69 metropolitan areas across USA and Canada and acquired relevant data to describe office, retail, and job concentrations within these regions. We then created 50 m grids for each metropolitan area and the acquired data. Using these populated grids, the unsupervised image segmentation model was performed, which resulted in the clustering of given grid cells.

Although some clusters showed similar features, we found clusters divided into discrete clusters due to spatial discrepancies. To solve this problem, the clusters were combined using the aggregate clustering method, which resulted in identifying one distinctive cluster in terms of commerce. In the identified cluster, some polygons where the value of variables was lower than our thresholds were removed. Afterwards, the captured polygons were aggregated into one file. This method resulted in 23,751 commercial boundaries across 69 selected metropolitan areas in USA and Canada. Furthermore, the commercial score of each boundary, which shows the comprehensive commercial properties of the area based on retail, office and job density, was calculated based on the commercial and geographical characteristics and Principal Component Analysis (PCA).

This study employed a multifaceted approach considering the retailer location as well as office and job agglomerations altogether, which offered a broader and more detailed and comprehensive perspective of major metropolitan areas’ distinct commercial areas in the USA and Canada. The developed boundary would be valuable for other researchers, governments, and other related stakeholders because the boundary not only enables the comprehension of the dynamics of retail geography in the areas but also can be employed as the base boundary for diverse studies related to retail, e.g., predicting the probability of retail gentrification^13^, measuring the recovery of commercial areas from the pandemic^4,14,15^, estimating the impact and resilience of commercial areas in the lockdown^16^, estimating the e-commerce vulnerability of commercial areas^17^, identifying the fiscal health of commercial areas^18^, etc. A detailed example of employing commercial boundary for the study is that researchers can count the types of retailers, including small local stores, boutiques and chain stores, based on the boundary, which enables estimating the impacts of the retail structure in terms of parking, public transportation, tax revenue, employment and more. Over time, it would be possible to track retail gentrification, gaps and vulnerabilities based on the retail structure changes. In addition, the output can also be employed potentially to enhance urban and regional planning strategies, ultimately organising cities into more economically efficient and vibrant hubs.

However, we must note that this study has some limitations. First, the boundary only covers some selected metropolitan areas. It also lacks consideration for post-pandemic job trends. In addition, the accuracy of commercial boundaries tends to depend on the completeness of OpenStreetMap (OSM), which varies based on the population of the area^19–21^. However, the probability of missing geographical entities on OSM tended to be minimised because we targeted the large metropolitan areas with abundant participants in OSM.

Nonetheless, the identified commercial boundaries are still valuable to describe the overall commercial geography in the area. Since we also employed open-access data and developed a series of analyses that can be easily followed, researchers and related stakeholders are able to reproduce and modify the data based on their needs, which underlines the usability of this study.

## Methods

### Study area

The first step in identifying commercial boundaries is choosing suitable study areas. The study area of this study is 69 major metropolitan areas across the USA and Canada. This is because, in the United States and Canada, populated areas are relatively limited compared to the extensive land area of the countries; with most of the population concentrated in cities and suburbs^22,23^, these areas tend to have much more clearly articulated commercial boundaries. In addition, there are diverse retail formats in the non-urban areas, such as farm-stands, which have grown in a different way than typical retailers^24,25^. These characteristics in rural areas and small towns are likely to increase the uncertainty in investigating commercial boundaries and to blur the estimated boundaries.

In practical terms, it is difficult to measure the commercial boundaries across the USA and Canada due to the limitation of available computing power. Indeed, generating 50 m grids covering both countries and training the deep learning model require significantly large computing power, which means that it is hard for individual researchers using laptops or desktop computers to achieve these goals. Therefore, we opted to focus on major metropolitan regions in the US and Canada.

To select the major metropolitan areas, Metropolitan Statistical Areas (MSA) and Census Metropolitan Areas (CMA) were employed for the US and Canada respectively. We chose the top 50 MSAs by population from 2019 data for the US, while for Canada, we selected the top 5 CMAs. In addition to the top metropolitan areas based on population, 9 MSAs in the United States and 5 CMAs in Canada were considered additionally based on the downtown boundaries of Downtown Recovery project^26^ at the School of Cities at the University of Toronto.

**Fig. 1:**
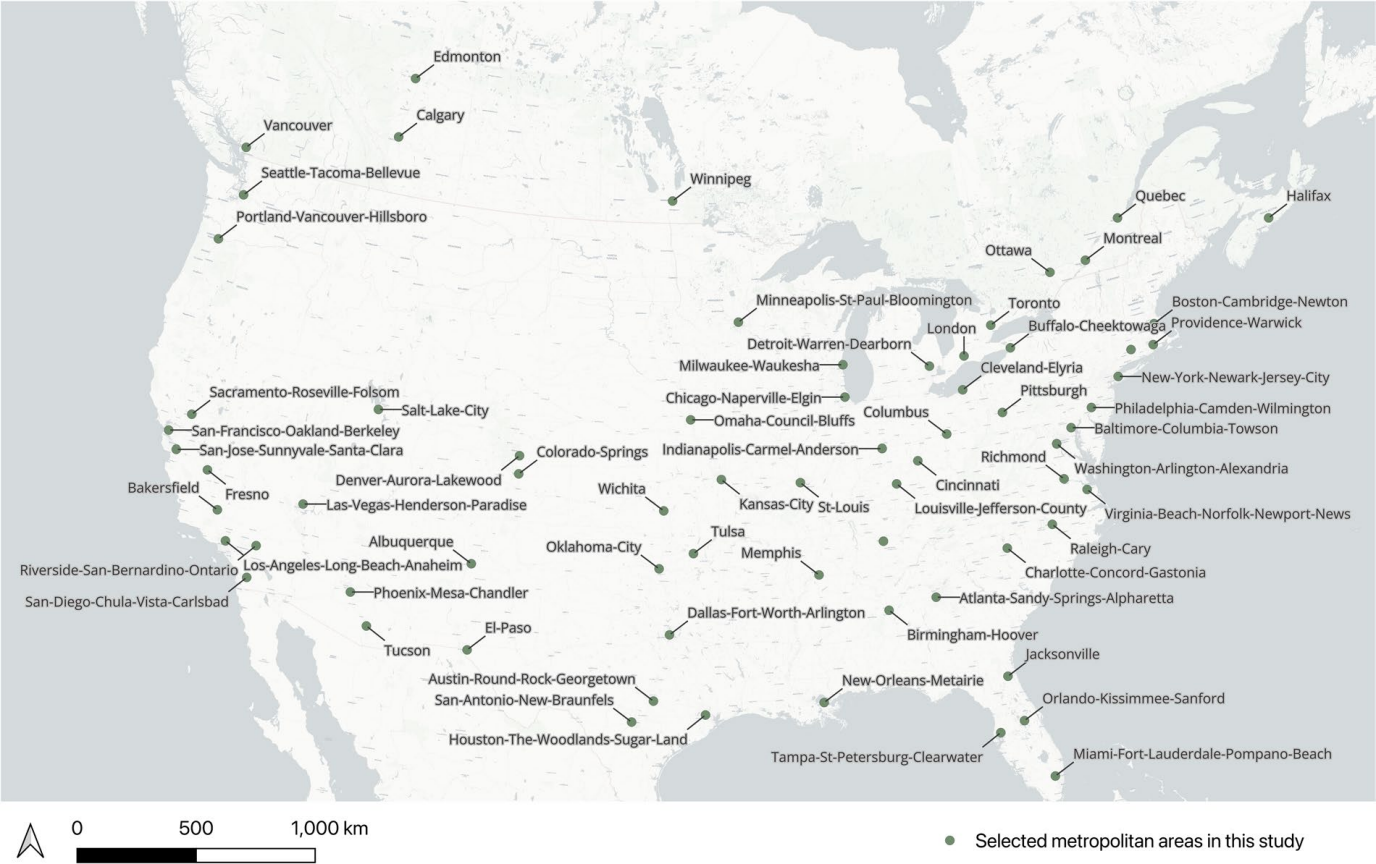
The geographical distribution of selected metropolitan areas (some metropolitan areas were modified) in USA and Canada.

69 metropolitan areas were selected in total. However, certain metropolitan areas required modifications due to the inclusion of extensive non-urban regions like deserts, national parks, and conservation areas. This is because for these metropolitan areas that include these regions, the generated grid was so large that it exceeded the available GPU capacity provided by Colab. This resulted that the too large size grids were not able to import the grid into the image segmentation model. For this reason, the non-urban regions in which commercial areas are not likely to be located were removed in some metropolitan areas by modifying the boundary of them. As a result, we manually adjusted these areas. Figure 1 depicts the final geographical location and boundaries of modified 69 metropolitan areas.

### Source data

To accurately measure the boundaries, it is necessary to employ proper datasets that can effectively represent the distribution of office, retail, and job densities. Despite the availability of an extensive array of private datasets providing detailed locations of offices and retail, these often prove inaccessible to individual researchers and stakeholders due to the considerably expensive cost. An additional merit of using open-source data is that the result of this study is readily replicable in other regions or at different time periods. Therefore, we employed open-source data, which offers valuable information about the locations of offices and retail, facilitated by OSM^27^. OSM data has been validated by estimating the accuracy of its attributes, such as location, shape, count, density, etc., through comparing reference datasets in a variety of case study areas^28,29^. Macdonald *et al*. successfully delineated the overall retail boundaries in Scotland and Northern Ireland using the OSM retailer data, even though they employed Valuation Office Agency (VOA) data for England and Wales^2^. In particular, their retail boundaries showed similar performance in Scotland compared with the ordnance survey high street boundaries. To estimate the OSM office data quality, the coverage rates were also estimated by comparing the differences between the area of OSM buildings tagged as ‘office’ and that of secured office properties supplied by CoStar^30^, a commercial real estate information company with the largest inventory. Specifically, Chicago-Naperville-Elgin, Omaha-Council Bluffs, Vancouver and Toronto were selected. Since the CoStar data was supplied in point format, we estimated the ratio between the total OSM building floor areas in which CoStar points were located and the total CoStar building floor areas in metropolitan areas. The coverage rates for the selected areas were 0.75, 0.55, 0.51 and 0.75 in order. Although the metropolitan areas varied the coverage rates, all case study areas recorded over 0.5, which shows that OSM data is reliable despite not as much as private company data. These characteristics of OSM likely guarantee the quality of OSM data for the selected metropolitan areas.

Table 1 represents specifically the employed datasets and their sources in order to describe the office, retail and job density. To obtain the office and retail data from OSM, Python and osmnx package^31^ were employed. For office representation, we obtained all buildings tagged as ‘offices’ from OSM. Moreover, we also downloaded some geographical entities that have an ‘office’ key with stated tags, namely ‘accountant’, ‘administrative’, ‘company’, etc. from OSM. OSM defines an office key as “A place of business predominately providing services”^32^. The criterion for selecting proper tags in the office key was whether or not they supply professional services associated with commerce. Table 2 represents the detailed query to obtain the office data.

**Table 1:** The detailed description of employed data with year and sources.

Variables	Data	Year	Source
Offices	Building with office tag	2023	OpenStreetMap^27^
	Office with selected tags		
Retail	Building with retail tag		
	Amenity and shop with selected tags		
Job Density	Workplace Area Characteristics in US	2019, 2018 (Mississippi)	LEHD, U.S. Census Bureau^33,35^
	Usual Place of Work in Canada	2016	Statistics Canada^34,36^

**Table 2:** The employed keys and tags to achieve office data from OSM in this study.

Key	Tag
{‘building’}	{‘office’}
{‘office’}	{‘accountant’, ‘government’, ‘company’, ‘construction_company’, ‘consulting’, ‘coworking’, ‘diplomatic’, ‘employment_agency’,‘financial’, ‘financial_advisor’, ‘government’, ‘it’, ‘lawyer’, ‘newspaper’, ‘ngo’, ‘tax_advisor’}

In the case of retail, the retailers and retail building data were obtained from OSM. Specifically, all geographical entities with a ‘shop’ key were downloaded, and some entities, which had an amenity key with some tags associated with our daily consumption behaviours such as ‘restaurant’, ‘café’, and more were employed. The buildings with ‘retail’ tag were downloaded and used as retail buildings. The detailed keys and tags are described in Table 3.

**Table 3:** The detailed keys and tags to obtain retail data from OSM in this study.

Key	Tag
{‘building’}	{‘retail’, ‘supermarket’}
{‘amenity’}	{‘restaurant’, ‘cafe’, ‘fast_food’, ‘bank’, ‘pharmacy’, ‘bar’, ‘pub’, ‘pub’,‘post_office’, ‘marketplace’, ‘nightclub’, ‘bureau_de_change’, ‘food_court’}
{‘shop’}	True

Lastly, Workplace Area Characteristics (WAC) data in 2019 for the US (except for Mississippi) from Longitudinal Employer-Household Dynamics (LEHD)^33^ in the U.S. Census Bureau and the usual place of work data in 2016 for Canada from Statistics Canada^34^ were employed to represent the concentration of jobs for the US and Canada respectively. Mississippi employed 2018 data due to the lack of 2019 data. The reason for employing 2019 job data is that 2020 data is unsuitable to represent the job density due to the pandemic that led to numerous layoffs and other disruptions. Although employing data in 2023 would reflect the new normal trends after the pandemic, this data is not yet available. This study used data in 2019 (for the United States) and 2016 (for Canada) to reflect normal job patterns. Both data represent the workplace location of workers aggregated at the census blocks in the United States and the Dissemination Area (DA) in Canada. As mentioned in the previous section, all kinds of jobs were employed in both countries because it is difficult not only to select which jobs related to the retail and office sector in detail but also to obtain detailed data. Since the obtained data lacked the geometry information, we joined the data with census block and DA downloaded from the U.S. Census Bureau^35^ and Statistics Canada^36^, respectively. In order to estimate the job density, the total job count from the data was divided by the area of boundaries, which led to building the job density data.

### Data pre-processing

After selecting proper variables from diverse sources, we performed a series of pre-processing steps on the retrieved data prior to estimating commercial boundaries in the metropolitan areas. As a first step, it is necessary to assign the variables to the grids to create matrices for the deep learning model. Before assigning data, it is crucial to investigate the optimal grid size that can capture the granular scale commercial distribution because the areal units impact the final output of the analysis sensitively^37^. To achieve this goal, we selected a diverse sample of case study metropolitan areas, namely ‘San-Francisco-Oakland-Berkeley’, ‘Toronto’, ‘Salt-Lake-City’, ‘Colorado-Springs’ and ‘Quebec’. We then generated 25 m, 50 m, 100 m and 500 m grids spanning each case study area and assigned the variables to the grids. Subsequently, we consolidated the variables of case study areas and estimated the overall standard deviation of each variable by each grid size. Since the higher standard deviation means excessive variability within the grid, it can hinder the homogeneity of identified commercial boundaries. Therefore, the lower the standard deviation is, the more optimised the grid size.

**Fig. 2:**
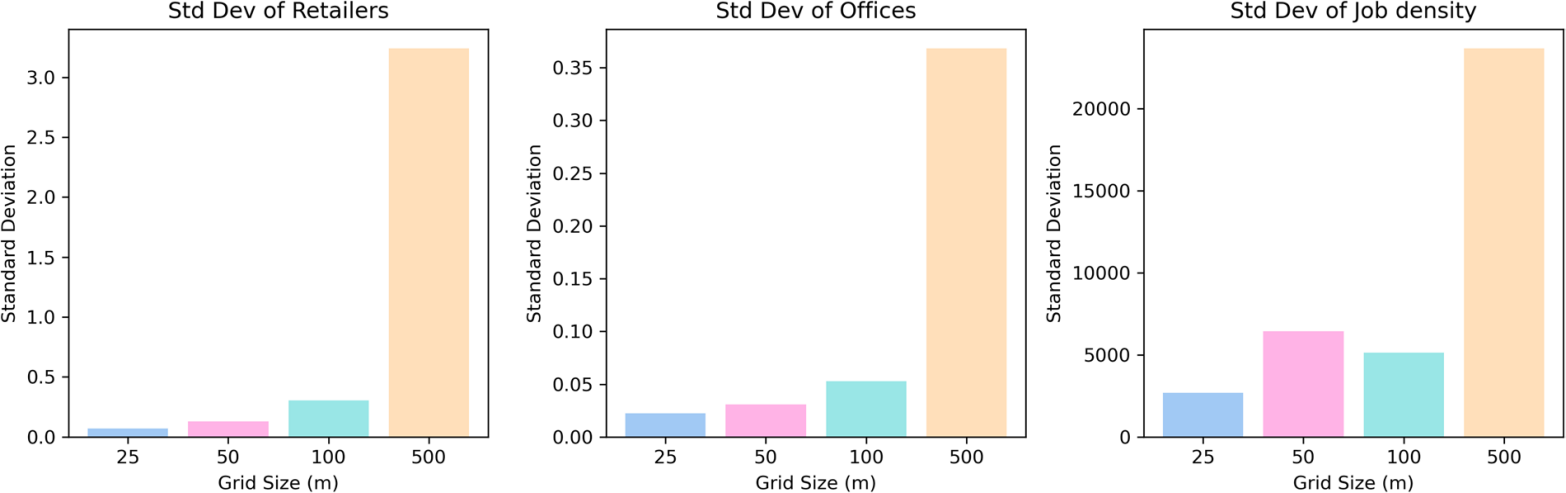
The changes in the standard deviation of retailers, offices and job densities by grid sizes.

Figure 2 represents the standard deviation of the variables by grid size. The results revealed that smaller grids tended to show a lower standard deviation for all variables except for job density. In the case of job density, the standard deviation of the 50 m grid was slightly higher than that of the 100 m grid. This result suggested that the smaller grids led to low variability within the grid and a high degree of consistency. Although the standard deviation result suggested a 25 m grid for this study, this grid size may complicate the processing and analysis of data and require substantial computing capabilities due to an excessive number of grids. These circumstances make it hard for individual researchers with limited computing power to estimate the boundaries. Moreover, since the standard deviation difference between the 25 m grid and the 50 m grid is not significant compared to the 50 m and 100 m differences in most variables, we selected a 50 m grid.

In addition to the statistical approach, we explored the previous studies to support 50 m grids. They argued not only that the tendency of people to shop at nearby locations leads to the agglomeration of retailers but also that 50 m can properly examine relationships between different retail locations^1,38–40^. This underlines that the 50 m grid is the optimal size for delineating commercial boundaries. Therefore, the *N* × *M* 50 m grids spanning each metropolitan area were generated on the basis of the geographical extent of the area.

This was followed by counting the selected variables based on the given grids, which resulted in the variables being aggregated at the 50 m grid level. Because the processed grid data were built in geospatial data format (specifically GeoParquet format), they were transposed into the matrix format in order to perform unsupervised image segmentation. Through this data pre-processing, 69 matrices representing the selected metropolitan areas were built.

The subsequent step involved a normalisation process for the selected variables not only to match the range of data but also to improve the training performance of the deep learning model. The normalisation range was set between 0.5 and 1. The reason for employing this range is that the variables—offices and retail—tended to be concentrated in specific areas, implying that numerous cells remained at 0. To clearly differentiate between areas with and without the presence of offices and retail, we employed a range of 0.5 to 1. Although the job density encompassed entire areas, the same range was applied to match the range of data with the other variables.

Due to the varying geographical extent of each metropolitan area, the resulting matrix shapes also differed. To train a deep learning model based on the given data, it is important to match the input shape of matrices. In this study, the random crop method, which crops the matrix at a specific size on a random location, was employed to create same-shaped matrices, and the decided crop size was 100 × 100. Initially, our aim was to generate 1000 random cropped matrices for each metropolitan area, but it risked creating overlapping matrices in relatively smaller areas. This posed a threat of over-training specific patterns of smaller metropolitan areas in the deep learning model. As a preventive measure, normalisation between 10 and 1000 depending on the matrix size of each metropolitan area was employed to adjust the number of random cropped matrices. The maximum metropolitan was New-York-Newark-Jersey-City, and the minimum metropolitan area was London. Ultimately, we generated 15,878 random cropped matrices for the unsupervised image segmentation, and for the prediction phase, the original matrix of each metropolitan area was employed.

### Unsupervised image segmentation model

The pre-processed data was then subjected to a series of analytical steps encompassing unsupervised image segmentation, and identifying commercial boundaries based on segmentation results. In the unsupervised image segmentation, we employed an unsupervised segmentation model developed by Kim *et al*.^41^. Although the original intended purpose of the model is for RGB image segmentation, its application in our study for commercial boundary identification remains viable considering that grid-based matrices with our selected variables bear a similar structure to RGB images. The structure of this model is organised by multiple layers combined with Convolution Neural Network, ReLU activation function and batch normalisation layers. The batch normalisation is employed to convert the *K* number of variables in each pixel into a singular value representing the cluster.

The most notable characteristic of this model is to simultaneously consider the similarity of attributes in the same cluster as well as spatial continuity of clusters. According to Kim *et al*.^41^, the estimation of similarity and continuity of clusters were measured by the developed loss function of the model. The comprehensive loss function is detailed below:

$$L={L}_{sim}\left(\left\{{{\boldsymbol{r}}}_{n}^{{\prime} },{c}_{n}\right\}\right)+\mu {L}_{con}\left(\left\{{{\boldsymbol{r}}}_{n}^{{\prime} }\right\}\right)$$

Where ${L}_{sim}\left(\left\{{{\boldsymbol{r}}}_{n}^{{\prime} },{c}_{n}\right\}\right)$ and ${L}_{con}\left(\left\{{{\boldsymbol{r}}}_{n}^{{\prime} }\right\}\right)$ represent the similarity and continuity loss, respectively. *μ* is a weight of continuity. The higher *μ* means that the model concentrates more on the spatial continuity of the clusters. *n* depicts each pixel in the given input *N*. ${{\boldsymbol{r}}}_{n}^{{\prime} }$ is normalised output, and *c*_*n*_ is the final clustering result based on the ${{\boldsymbol{r}}}_{n}^{{\prime} }$ using argmax function.

The similarity loss function is measured through the CrossEntropyLoss between $\left\{{{\boldsymbol{r}}}_{n}^{{\prime} }\right\}$ and {*c*_*n*_}, which leads the model to learn in the direction of increasing the similarity of the cluster. In the case of the continuity loss function, it is calculated using the following equation:

$${L}_{con}\left(\left\{{{\boldsymbol{r}}}_{n}^{{\prime} }\right\}\right)=\mathop{\sum }\limits_{\xi =1}^{W-1}\mathop{\sum }\limits_{\eta =1}^{H-1}\left\Vert {{\boldsymbol{r}}}_{\xi +1,\eta }^{{\prime} }-{{\boldsymbol{r}}}_{\xi ,\eta }^{{\prime} }\right\Vert +\left\Vert {{\boldsymbol{r}}}_{\xi ,\eta +1}^{{\prime} }-{{\boldsymbol{r}}}_{\xi ,\eta }^{{\prime} }\right\Vert $$

Where *W* and *H* are the shapes of given input matrix. ${{\boldsymbol{r}}}_{\xi ,\eta }^{{\prime} }$ represent the output value of ${{\boldsymbol{r}}}_{n}^{{\prime} }$ at (*ξ,η*) location. This loss function can control the excessive number of clusters.

### Fine tuning of hyperparameter

With the unsupervised image segmentation model and randomly cropped matrices in place, the fine-tuning of hyperparameter process was performed. Given the large volume of the training dataset (approximately 16,000 random cropped matrices), training the segmentation model using all training data simultaneously proved challenging. Therefore, it was necessary to employ the mini-batch training, which partitioned the entire training data into manageable batch sizes, thereby enabling the deep learning model to learn gradually. In this study, the batch size was set at 32.

It was followed by the hyperparameter tuning process using a grid-search method that trains the model using the possible hyperparameter combinations and subsequently measures the performance based on the hyperparameter combinations. Although Kim *et al*.^41^ employed the mean of Intersection Over Union (mIOU) method—a comparison of the overlap ratio between ground truth and clustering result—to estimate the performance of the segmentation model, it is hard to use it in this study because of the unavailability of ground truth data in our study. Instead of mIOU, we employed the final loss value of each hyperparameter combination to estimate the performance of the model. This is because the better the trained model, the less the loss value will be. Key parameters used included the number of iterations, learning rates and *μ* (the weight of spatial continuity), and their ranges were {3,4,5}, {0.1,0.01,0.001} and {5,10,50} respectively. Based on these hyperparameter combinations, the model was trained using the same training data, and eventually, we identified the most suitable hyperparameter combination—the combination of 5 iteration numbers, 0.001 learning rates and 10 *μ*—with the lowest loss value (1.73).

Thereafter, the clustering was performed using the trained (equipped with the best hyperparameter combination) and the prediction dataset, which contained the full matrices of all metropolitan areas. Consequently, there were 58 clusters spanning the entire metropolitan region.

### Extracting commercial boundaries

Although the unsupervised image segmentation model successfully clustered the specified metropolitan areas, producing 58 clusters, a subsequent series of analyses was required to extract commercial boundaries from the clustering result due to the results representing segments of metropolitan areas. Therefore, it was crucial to establish which clusters constituted commercial areas. The additional problem of the clustering result is that clusters showing similar features were classified as different clusters due to the spatial distance. For this reason, they need to be considered as the same cluster. To address these challenges, a framework to identify commercial boundaries from the clustering results was developed.

The first step of this framework is to remove the clusters where the count of offices and retailers was 0, for efficacy in implementing the framework. As a result, the 23 clusters were eliminated among 58 clusters. This was followed by re-clustering the output clusters based on their attributes. Since the spatial continuity of clusters could have been obtained through the image segmentation model, it was necessary to merge the clusters with similar attributes. To achieve this goal, agglomerative clustering, which merges similar clusters, and silhouette scoring were employed, which allowed us to conduct re-clustering with an optimal number of clusters. The former is based on hierarchical clustering, which can conduct the clustering with a bottom-up approach^42^. Silhouette score was employed to find the optimal number of the clusters. A silhouette score closer to 1 indicates optimal clustering. We conducted the re-clustering process by changing the number of clusters from 2 to 34, measuring the silhouette score for each result. It was noted that the score reached a maximum value of 0.96 when the number of clusters was 2. According to the re-clustering result, all clusters except for cluster labelled ‘57’ were aggregated as one cluster.

We eliminated commercial boundaries that were significantly small in size or whose variables (office and retail) were below the established threshold. In the case of the boundary size criterion, we designated 2500 m^2^—equivalent to one grid cell—as the threshold size. The threshold of offices and retailers was 5 and 10 establishments, respectively. In the case of retail threshold, we used the typical value in the previously studies^1,2,43^. On the other hand, for offices, there were no previous studies, so we based the threshold on the distribution of offices. Consequently, the metropolitan area in which the largest number of commercial boundaries was identified was an area covering Los Angeles Long Beach Anaheim with 1662 commercial boundaries, whereas there were only 43 commercial boundaries in the Halifax area.

**Fig. 3:**
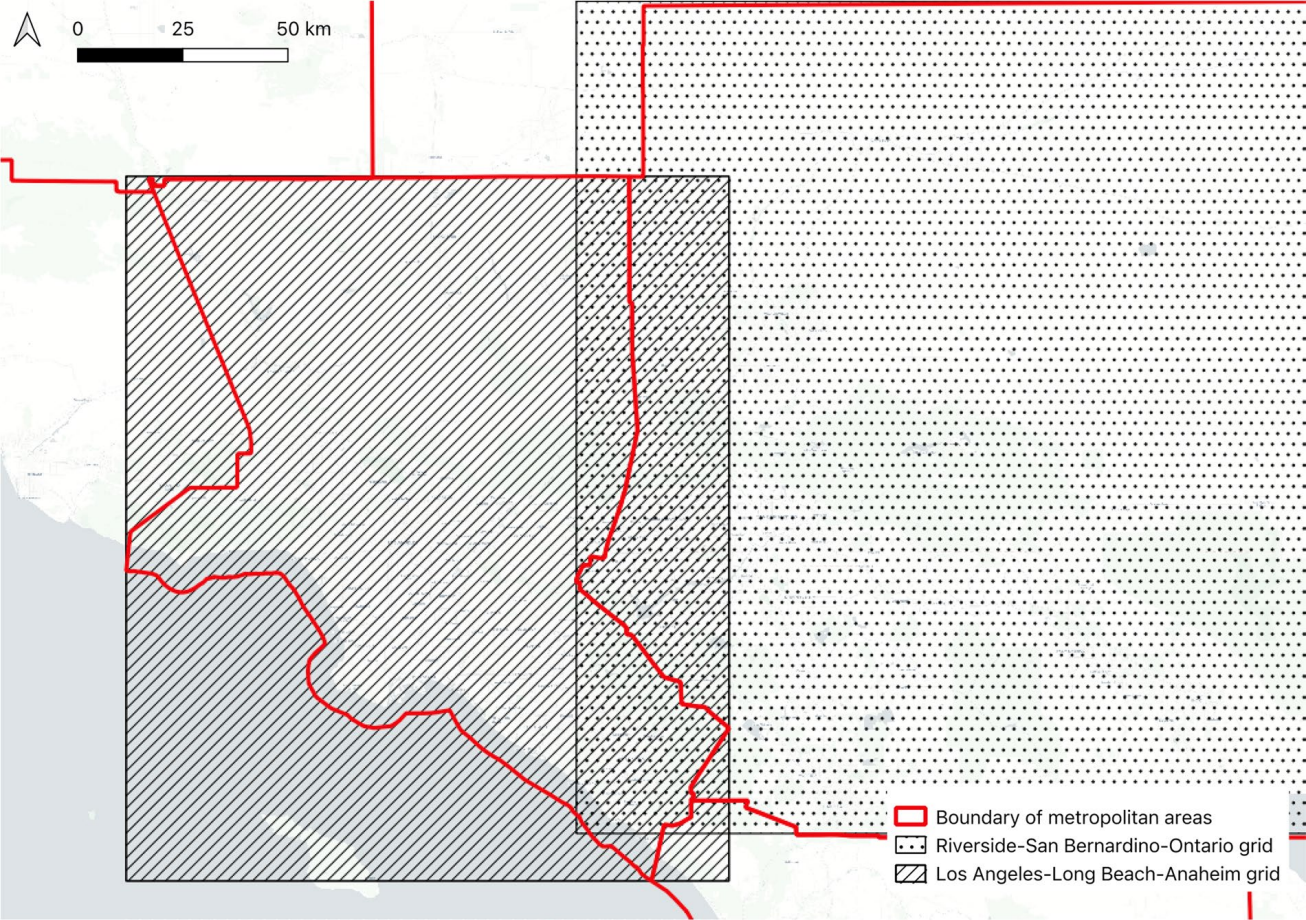
The example of touched metropolitan areas and their grids. The red line represents the boundary of metropolitan areas. The hashed and dot box represent the Los Angeles-Long Beach-Anaheim and Riverside-San Bernardino-Ontario grids respectively.

The estimated commercial boundaries by the metropolitan areas were aggregated into one shapefile. Since some metropolitan areas were touched, which meant that the created grid based on the geographical extent of the areas was highly likely to overlap. For example, the Los Angeles-Long Beach-Anaheim metropolitan area is touched with Riverside-San Bernardino-Ontario, San Diego-Chula Vista-Carlsbad and Bakersfield (see the red line in Fig. 3). Since the grid needs to be squared shape, the extent of grid covering Los Angeles-Long Beach-Anaheim (see hashed box in Fig. 3) invade the other metropolitan area. Likewise, the grid boundary of Riverside-San Bernardino-Ontario (see the dot box in Fig. 3) also invades the Los Angeles-Long Beach-Anaheim area. As a results, there is an overlapped area between two grids. This resulted in the existence of duplicated commercial boundaries. Therefore, to deal with these cases, we identified the overlapped commercial boundaries and compared the size of the boundaries. The larger one remained and the smaller one was removed. Based on this principle, all commercial boundaries were merged, and 23,751 boundaries were finally identified.

In addition, the names of metropolitan areas and states or provinces were assigned to the corresponding commercial boundaries. This was achieved by intersecting commercial boundaries and metropolitan areas; if the commercial boundaries were located within the metropolitan areas, the name and state/province of these areas were assigned accordingly. Nonetheless, if a commercial boundary was situated outside the metropolitan area due to a discrepancy between the *N* × *M* 50 m grid and the actual metropolitan boundary, we affiliated it with the nearest metropolitan area. This was also followed by assigning the address of the commercial boundary through the reverse geocoding method. Specifically, we measured the centroid of each commercial boundary and performed reverse geocoding using Python and Geopy package^44^. The achieved street, hamlet (for the US) or neighbourhood (for Canada), city and state names were employed as the address of the boundaries.

**Fig. 4:**
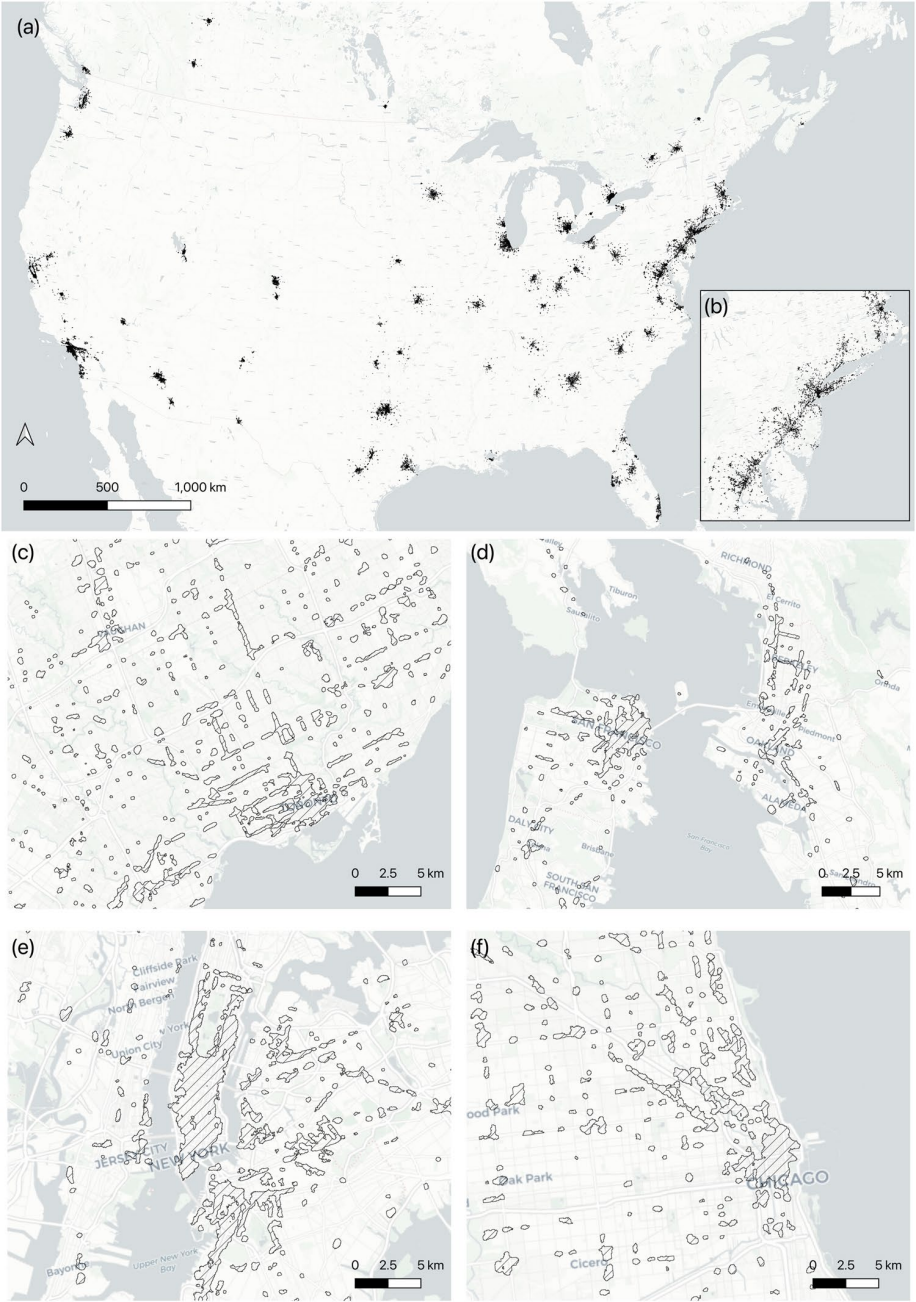
The overall distribution of merged commercial boundaries in the selected metropolitan areas (**a**). (**b**) represents the detailed description of commercial boundaries located in the megalopolis area. From (**c**) to (**f**) show the overall distribution of commercial boundaries in Toronto, San Francisco, New York and Chicago.

This series of analyses culminated in the creation of the commercial boundary data. Figure 4a describes the overall distribution of commercial boundaries in the 69 metropolitan areas across USA and Canada. It is notable that the commercial boundaries in the Northeast megalopolis, extending from Boston to Washington D.C., appear almost continuous, emphasising the conurbation of the area (Fig. 4b). Furthermore, Fig. 4c,f depict more detailed boundaries in some notable metropolitan areas, namely Toronto, San Francisco-Oakland, New York and Chicago areas. The geographical distribution pattern of commercial boundaries in those regions shows a tendency for small and medium-sized commercial boundaries connected as linear corridors on the basis of central large-size commercial boundaries.

### Estimating commercial scores

**Fig. 5:**
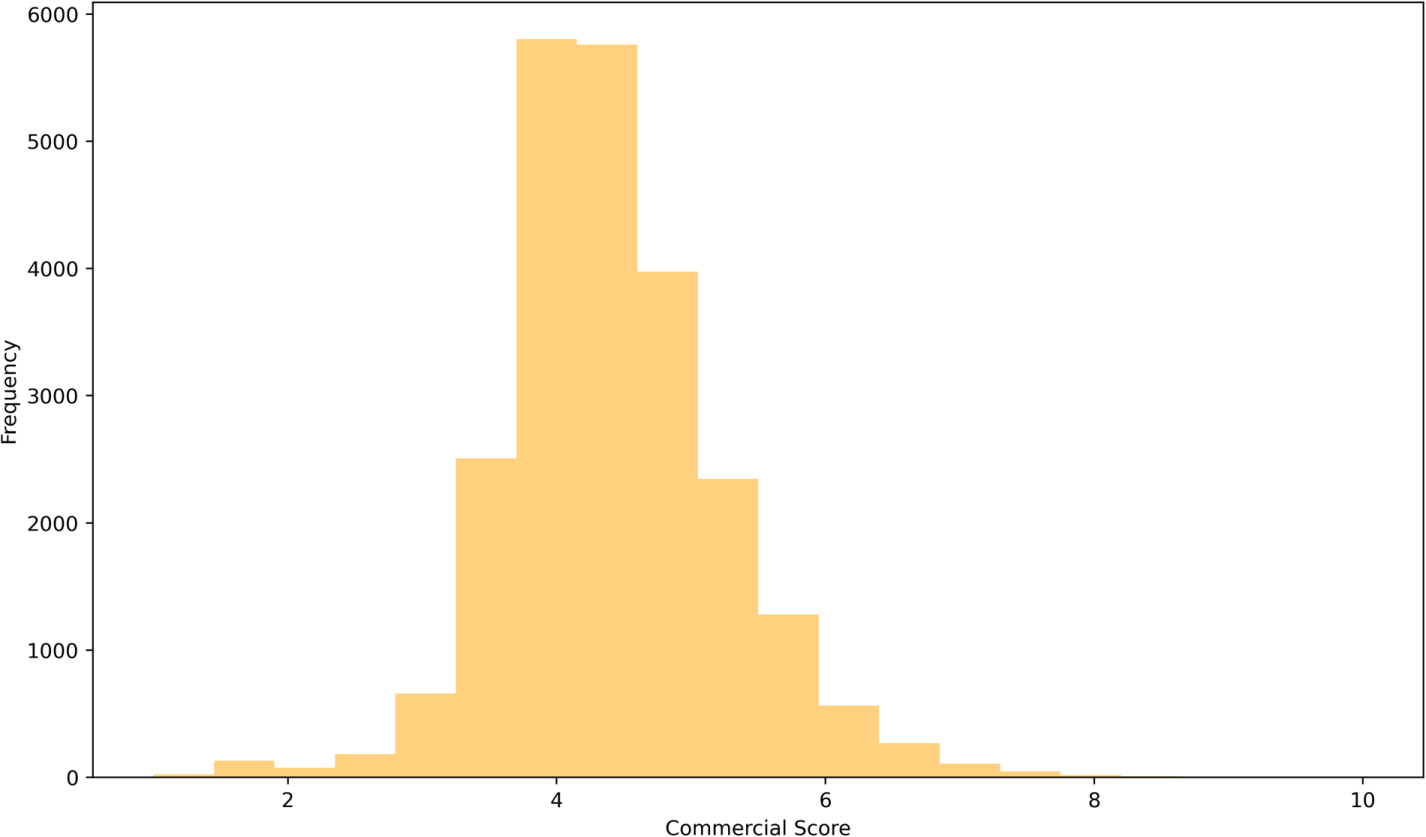
The overall distribution of commercial score.

After identifying the commercial boundaries, we estimated the commercial scores based on the retail, office and job properties of each boundary, which enabled us to improve the applicability of this dataset. This score represents the relative concentration of office, retail and job density of each commercial boundary. To achieve this goal, we employed Principal Component Analysis (PCA). This method is widely employed not only for dimension reduction of multivariable datasets but also for integrated scoring of multiple variables, maintaining as many of the characteristics of data as possible^45^. However, since the assumption of PCA is a normal distribution^13^, we performed a logarithm and standardisation for the variables in order to convert the distribution variables to follow a normal distribution and match their range. Based on the converted data, the PCA was conducted, which resulted in a comprehensive score in terms of offices, retails and job density. As a result, the score was derived for each commercial boundary, and the range of the score was from −5.2 to 8.4. The higher the values of the variables, the higher the PCA score. However, the negative value makes it hard to understand the score of commercial boundaries intuitively. For this reason, we employed normalisation with the range of 1 to 10 to adjust the score range. The mean and median commercial scores were 4.4 and 4.3, respectively. The address of the commercial boundary recording the highest score is 5th Avenue, City of New York, New York, United States, with a 10 score, whereas the address of the lowest commercial boundary is Bethridge Road, Highfield, Toronto, Ontario, Canada, with 1. Figure 5 represents the overall distribution of commercial scores. The distribution tends to follow the normal distribution and most values are concentrated between 4 and 5.

## Data Records

The output of this study is commercial_boundaries.gpkg, and the coordinate system is EPSG:3857. Table 4 describes the detailed column names and their descriptions. In detail, the boundary data contains the ‘CB_ID’ representing their intrinsic ID and Area (m2) depicts the area of commercial boundaries. ‘MSA_NAME’ and ‘MSA_ID’ describe the names of metropolitan areas and their ID. For the ID, we employed the ‘GEOID’ for the US and ‘CMAUID’ for Canada as the ‘MSA_ID’. ‘STATE/PROVINCE’ shows the states/province code in which the commercial boundaries are located respectively. If the metropolitan areas are across multiple states or provinces, the ‘STATE/PROVINCE’ indicates all corresponding states/provinces. For instance, STATE/PROVINCE code for commercial boundaries located in Ottawa metropolitan areas is ON-QC. Lastly, the ‘Commercial_score’ is the previously estimated commercial score of each commercial boundary, representing the relative concentration of office, retail and job density to improve the applicability of data.

**Table 4:** The detailed description of column names in the commercial_boundaries.gpkg file.

Column names	Type	Description
CB_ID	Integer	The assigned intrinsic ID of commercial boundaries
Area(m2)	Float	The area of commercial boundaries
MSA_NAME	String	The name of metropolitan areas belonging the commercial boundaries
MSA_ID	String	The code of metropolitan areas belonging the commercial boundaries
STATE/PROVINCE	String	The state/province of the metropolitan areas
Commercial_score	Float	The estimated commercial score of the commercial boundaries

This data is available in Figshare^46^ or the School of Cities GitHub (https://github.com/schoolofcities/commercial-boundaries).

## Technical Validation

It is important to compare the identified commercial boundaries and previously generated areas in order to validate the commercial boundaries. For the validation process of these commercial boundaries, we employed several datasets, including the CDRC US Retail Centre Boundaries^1,38^, downtown boundaries based on job density^4^^,^ and Commercial Centre (CC) and Central Business Districts (CBD)^5^. While the downtown, CC, and CBD boundaries cover only select major cities in major metropolitan areas, the US retail centre data encompasses the entire United States territory. Since our boundaries were built based on the selected major metropolitan areas, the US retail centres within the geographical extent of the built *N* × *M* 50 m grid were selected in the validation process.

The validation process involved clipping the commercial boundaries using each boundary dataset, given the varying geographical coverage of these boundaries. Subsequently, we measured the coverage ratio between the area of the clipped commercial boundary and each validation boundary employed. Estimating coverage ratio is one of the widely employed methods for validation (e.g. Macdonald *et al*., compared with OS High Streets and their boundaries^2^, and Pavlis *et al*., compared with Geolytix boundaries and developed retail extent^43^). Table 5 represents the statistics of employed validation boundaries and their coverage ratio. The validation results revealed that the CBD developed at Brookings Institution had the highest coverage ratio at 0.54. The comparison results with CDRC US retail centres yielded a ratio of 0.4, even though it covers significantly larger areas than other validation boundaries. It is followed by CC and downtown boundaries. Although directly comparing the developed commercial boundaries and previously established validation boundaries can be challenging due to differences in definition and data sources, it can be seen that there are some areas that overlap with validation boundaries, while many others do not.

**Table 5:** The statistic of validation bounds and commercial boundaries with coverage ratio.

Validation Boundary	Area of validation bound (km^2^)	Area of clipped commercial boundaries (km^2^)	Coverage ratio
US Retail Centre	8163.46	3263.5	0.40
Downtown	897.68	177.08	0.20
Commercial Centre	933.88	241.28	0.26
Central Business Districts	157.94	84.68	0.54

For a more detailed validation of the commercial boundaries, the validation process was conducted at the city level (for downtown, CC and CBD) or metropolitan area level (for US retail centre). Table 6 provides information on the minimum and maximum coverage of each validation boundary, along with the names of cities or metropolitan areas with minimum and maximum values. In the case of the US retail centre comparison, the Seattle-Tacoma-Bellevue and Las-Vegas-Henderson-Paradise areas recorded maximum and minimum coverage ratios of 0.6 and 0.22, respectively. However, the remaining validation boundaries exhibited considerable variation. For instance, the coverage ratio for San Jose downtown was 0.81, while for Bakersfield, it was only 0.0002. The boundaries developed by the Brookings Institution showed a similar tendency to the downtown result. However, it is remarkable that the coverage ratio of the CBD in New York reached 0.96, which means that the CBD and commercial boundaries in this city are highly similar.

**Table 6:** The minimum and maximum coverage ratio with the name of cities and metropolitan areas by validation bounds.

Validation Boundary	Minimum (Name)	Maximum (Name)	Standard Dev.
US Retail Centre	0.22 (Las-Vegas-Henderson-Paradise)	0.6 (Seattle-Tacoma-Bellevue)	0.09
Downtown	0.0002 (Bakersfield)	0.81 (San Jose)	0.2
Commercial Centre	0.06 (Birmingham)	0.68 (New York)	0.15
Central Business Districts	0.05 (Birmingham)	0.96 (New York)	0.25

**Fig. 6:**
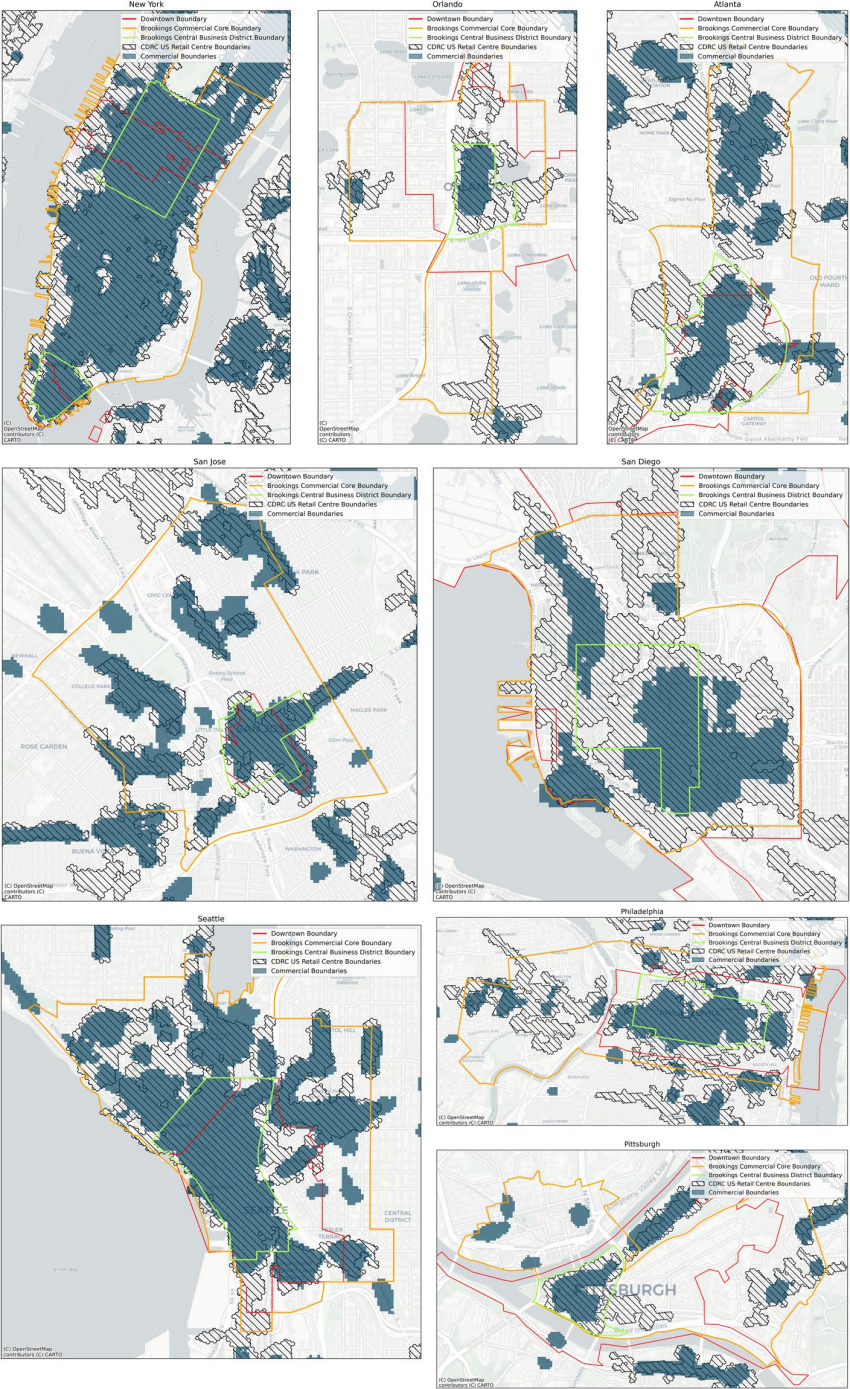
The geographical extent of the validation bounds and commercial boundaries in some selected cities.

To visually validate the commercial boundaries, we created maps for selected cities, which can show the geographical distribution of validation bounds and identified commercial boundaries (Fig. 6). These mapping results not only visually validate the accuracy of commercial boundaries but also reveal that commercial boundaries tend to capture more commercially distinctive areas than the validation boundaries. Specifically, since downtown and Brookings boundaries used the ZIP Code Tabulation Areas and census block respectively, whereas the commercial boundaries employed 50 m grids, the latter can capture detailed distinctive areas. In addition, compared with CDRC US retail centres, the advantage of commercial boundaries is that it is a more comprehensive approach to identifying the commercially distinct areas; it also allows the investigation of commercial boundaries in Canadian metropolitan areas. Although CDRC US retail centres considered retail, services (Depository Credit Intermediation, Insurance Carriers, Automotive Repair and Maintenance, and Personal Care Services), and leisure POIs to identify the boundaries^1,38^, diverse commercial activities were not represented. In particular, the commercial boundaries established in this study encompassed not only the offices and services related to daily life but also professional and admin services. Therefore, the commercial boundaries incorporated office, retail and job density, which enabled us to identify more detailed commercially distinguishing areas compared to validation boundaries employing only retailer agglomeration or jobs.

Consequently, the overall difference between the commercial boundary and the other validation boundaries tended to be caused by the difference in granularity of the employed geographical boundary as well as the variables, which does not mean that the measured boundary is not accurate. In particular, according to the visual validation, these results rather underlined that the commercial boundary identified the more conservative and arguably precise boundary of commercial areas than the other boundaries even though the aim and definition of each validation boundary is different.

However, the key limitations of this boundary are that, first, the dataset only covers the major metropolitan areas in the US and Canada. Although it is rational to focus on the population-concentrated areas to reduce uncertainty and confusion, the coverage of commercial boundaries would slightly reduce the usefulness of this dataset. In addition, this boundary is likely to depend on the completeness of employed OSM data. Specifically, the missing entities in OSM are highly likely to impact the delineated commercial boundaries, an inevitable limitation of this study. In the case of job density, we employed all kinds of jobs, whether or not they are related to the retail and office sectors. If the jobs related to the commercial boundary definition were employed, it would be likely to capture the boundaries more accurately. The other limitation of this dataset is that the job data does not reflect job distribution trends in the post-pandemic era due to the lack of updated data; this result in the differences between the office and retail data (based on 2023 data) and the job density data (based on 2019 data). Lastly, the boundary is limited in terms of qualitative validation due to the lack of input by local experts and related local stakeholders.

Despite these limitations, the estimated boundary in this study describes significantly granular and accurate commercial areas in the selected metropolitan areas. Considering that the completeness of OSM data relies on the population of the area, it could minimise the missing entity problem because we employed major metropolitan areas in this study. Lastly, commercial boundaries can partly take into account the post-pandemic conditions, as two of the three employed variables reflect the latest trends. As such, the generated commercial boundaries can be widely employed by other researchers as well as related stakeholders despite these limitations.

## Data Availability

The codes used in this study are available from Figshare^46^ or the School of cities GitHub (https://github.com/schoolofcities/commercial-boundaries). In addition, modified metropolitan areas, a saved Pytorch state file and commercial boundary result data can also be obtained from those pages. Readers can easily replicate the identified commercial boundary using the given codes and datasets.
